# Predicting therapeutic response to oral ketamine for chronic suicidal ideation: a Bayesian network for clinical decision support

**DOI:** 10.1186/s12888-020-02925-1

**Published:** 2020-10-28

**Authors:** Denise Beaudequin, Adem T. Can, Megan Dutton, Monique Jones, Cyrana Gallay, Paul Schwenn, Cian Yang, Grace Forsyth, Gabrielle Simcock, Daniel F. Hermens, Jim Lagopoulos

**Affiliations:** Thompson Institute, University of the Sunshine Coast, Locked Bag 4 (ML59), Maroochydore DC, QLD 4558 Australia

**Keywords:** Ketamine, Suicidal ideation, Bayesian network, Complex system, Response, Predictive modelling, Clinical trial

## Abstract

**Background:**

The glutamatergic modulator ketamine has been shown to result in rapid reductions in both suicidal ideation (SI) and depressive symptoms in clinical trials. There is a practical need for identification of pre-treatment predictors of ketamine response. Previous studies indicate links between treatment response and body mass index (BMI), depression symptoms and previous suicide attempts. Our aim was to explore the use of clinical and demographic factors to predict response to serial doses of oral ketamine for chronic suicidal ideation.

**Methods:**

Thirty-two participants completed the Oral Ketamine Trial on Suicidality (OKTOS). Data for the current study were drawn from pre-treatment and follow-up time-points of OKTOS. Only clinical and sociodemographic variables were included in this analysis. Data were used to create a proof of concept Bayesian network (BN) model of variables predicting prolonged response to oral ketamine, as defined by the Beck Scale for Suicide Ideation (BSS).

**Results:**

The network of potential predictors of response was evaluated using receiver operating characteristic (ROC) curve analyses. A combination of nine demographic and clinical variables predicted prolonged ketamine response, with strong contributions from BMI, Social and Occupational Functioning Assessment Scale (SOFAS), Montgomery-Asberg Depression Rating Scale (MADRS), number of suicide attempts, employment status and age. We evaluated and optimised the proposed network to increase the area under the ROC curve (AUC). The performance evaluation demonstrated that the BN predicted prolonged ketamine response with 97% accuracy, and AUC = 0.87.

**Conclusions:**

At present, validated tools to facilitate risk assessment are infrequently used in psychiatric practice. Pre-treatment assessment of individuals’ likelihood of response to oral ketamine for chronic suicidal ideation could be beneficial in making more informed decisions about likelihood of success for this treatment course. Clinical trials registration number ACTRN12618001412224, retrospectively registered 23/8/2018.

## Background

Suicide remains a leading and preventable cause of death globally [1]. In Australia, it is estimated that 3046 people died from intentional self-harm in 2018 [2]. Yet, to date, there is no definitive pharmacological treatment for individuals with chronic suicidal ideation (SI). SI is defined as “thoughts about self-harm, with deliberate consideration or planning of possible techniques of causing one’s own death” [3]. Current biological approaches to treating SI include antidepressants [4], lithium [5], clozapine [6], and electroconvulsive therapy [7]. However, these treatments have limitations. For instance, despite the range of pharmacological options, approximately half of patients remain treatment resistant even after months of use and continue to experience ongoing SI [8]. Further, selective serotonin reuptake inhibitors and serotonin and norepinephrine reuptake inhibitors, usually first-line antidepressant treatments, require 3–8 weeks to improve mood [8, 9], leaving individuals at greater risk for SI [10]. This highlights the need for new approaches.

### Ketamine treatment in suicidal ideation

The N-methyl-D-aspartate receptor antagonist ketamine, a widely used general anaesthetic and short-acting analgesic agent, has increasingly been used to treat chronic suicidal ideation. Ketamine treatment has been shown to result in rapid reductions in both SI and depressive symptoms in several clinical trials [11–13]. However, while ketamine is considered a promising treatment for patients with treatment-resistant depression and SI, individual patients vary in their response to ketamine [14, 15], and some individuals do not respond sufficiently, even with serial, titrated treatments. The reasons for the heterogeneity of response to ketamine are unclear but may reflect differences in individuals’ underlying demographic attributes, pathophysiology, genetic makeup, and clinical features [10]. The ability to systematically estimate the likelihood of a positive response to ketamine on an individual basis would be beneficial to clinicians and researchers. Identification of pre-treatment predictors of response, such as clinical, phenotypic and biological variables, will provide significantly improved clinical outcomes for patients and represent an opportunity for personalised healthcare provision [10]. Prediction analyses have the potential to provide insights into treatment candidacy and personalised treatment selection for suicidal individuals.

The objective of this study was to develop a simple, practical method of predicting the likelihood of sustained therapeutic response to serial doses of oral ketamine for chronic SI.

Using Bayesian network (BN) methodology, we combined demographic variables with pre-treatment clinical symptoms measured by clinical rating scales, to evaluate their potential to predict sustained response following the treatment course. We hypothesised that these factors, considered in unison, were eligible predictors of treatment response, defined as reduction in SI following a course of ketamine treatment in patients with chronic SI. The ensuing model forms the basis of a decision support system to augment clinical decision making.

### Predictors of response to ketamine

While numerous studies have aimed to identify predictors of ketamine’s treatment effect in treatment-resistant depression [10, 16–19], there appear to be fewer studies investigating predictors of ketamine response in chronic SI. Predictors such as positive family history of alcohol dependence [16], higher body mass index (BMI) [17, 18], pre-treatment anxious depression [20] and lower ratio of slow wave sleep activity [21] have been found to correlate with greater antidepressant response to a single dose of ketamine. In their study of clinical predictors of ketamine response in treatment resistant depression, Niciu, Luckenbaugh [17], found significant correlations between change-from-baseline Hamilton Depression Rating Scale and higher BMI, family history of alcohol use disorder and prior history of suicide attempt(s). Rong, Park [10] undertook a narrative review to identify multiple pre-treatment predictors of response to ketamine in treatment-resistant depression. Predictors included clinical variables (BMI, history of suicide attempts, family history of alcohol use disorder), neurochemical variables (glutamine/glutamate ratio), neuroimaging variables (anterior cingulate cortex activity), and indicators of cognitive functioning (processing speed). High BMI and a positive family history of alcohol use disorder were the most replicated predictors of treatment outcomes in this review. Del Sant, Magalhães [19] conducted a literature review to explore clinical characteristics predicting response to ketamine. In this study the sociodemographic variables of positive family history of alcohol abuse disorder in a first-degree relative, higher BMI and negative history of suicide attempts were associated with improvements in depression scores post- ketamine infusion. Ballard, Yarrington [15] developed data-driven groups of SI trajectories after ketamine administration, pooled from five clinical ketamine trials. This study evaluated clinical, demographic and neurobiological factors that might predict SI response to ketamine and found that a longstanding history of chronic SI and self- injury were associated with non-response to ketamine. These results emphasised the heterogeneity of SI response to ketamine and the potential independence of SI response from changes in depressed mood.

### Novel statistical methods for inference and prediction

Datasets used in psychiatric research now typically contain thousands of variables from multiple sources. Advances in neuroimaging, electroencephalograms and other multimodal sensing technologies have provided researchers with unprecedented capabilities for characterising subsyndromal and pathologic processes - and responses to interventions - in psychiatry, neuroscience and psychology [22, 23]. Such technologies provide volumes of data that exceed the capacity of classical statistical inference methods. Novel statistical methods are required to weigh the predictive power of multiple variables, especially clinical and demographic characteristics, in studies with limited samples [17]. Machine and statistical learning are being proposed as solutions to the ‘21^st^ century psychiatry’s information overload problems’ [23].

#### Bayesian networks (BN)

BN are an analytic methodology at the interface of artificial intelligence and statistics and are increasingly being recognised as a useful tool for diagnosis and prediction in complex systems such as health domains [24]. Based on probability distributions, BN are powerful risk assessment tools, particularly valuable for reasoning under uncertainty [25, 26], as they provide a transparent evidence base for visualising risk pathways and predicting outcomes. Importantly, Bayesian methods are able to produce reasonable results even with small to moderate sample sizes, especially when strong, defensible prior information is available [27, 28].

A BN is a directed acyclic graph in which the variables, represented as nodes, are connected by a directed arc implying causality [25, 26, 29]. Chance nodes have a number of user-defined ‘states’ that can be qualitative or discrete (e.g., ‘True/False’, ‘High/Low’, ‘> 5/≤ 5’). Conditional probabilities are assigned to each state, and algorithms compute the joint probability distribution of the network. Once quantified, BN can simulate multiple risk pathways, and intervention or exposure scenarios, providing the conditional probability of target outcomes. This particularly appealing feature of BN to simulate a range of scenarios is achieved by varying the evidence entered into the network. Thus, BN can be used to compare relative risks and examine concomitant interactions between variables, uncover relationships that may not have been apparent, compute the strength of associations between variables, and identify sensitivities for target nodes. The BN instantly updates when new information is provided and presents outcomes and influences in a straightforward manner to users from any discipline, via a convenient visual platform.

BN offer a systems approach to modelling risk and supporting decision making in complex domains such as psychiatry and medicine [25, 29]. This boasts several advantages over regression-based methods, in addition to easy transformation into decision support models. Arora et al. (2019) describe BN as knowledge engineering and machine-learning tools for individual-level risk estimation in health science, or ‘precision medicine’. For example, Bilek and Karaman [30] used BN modelling to investigate the relationships among psychiatric, demographic and socio-economic variables and psychometric scales such as the Beck Depression Inventory. In a clinical trial, Cleophas and Zwinderman [31] compared traditional statistical methods (unpaired t-tests, simple and multiple linear regressions) and machine learning (BN methods) in evaluating the efficacy of pravastatin on the decrease of low-density lipoprotein cholesterol, concluding that the machine learning methods provided better sensitivity of testing and were more informative. They found that BN were very efficient in describing multivariate distributions, in addition to being robust with respect to overfitting. BN have been used with different aims in neuroscience, including discovering associations between variables, performance of probabilistic reasoning over the model, and classification of new observations, with and without supervision [32]. In the current study, we propose a BN to predict the likelihood of therapeutic response to serial, weekly oral ketamine treatments in reducing chronic SI. This proof-of-concept model, with further development and validation, has the potential to form the basis of a clinical decision support system, with the aim of more efficient identification of treatment candidacy.

## Method

### Study design and setting

This study used data collected from the Oral Ketamine Trial on Suicidality (OKTOS; ACTRN12618001412224) exploring the use of oral ketamine for participants with chronic SI conducted at a single centre between August 2018 and October 2019. Although acute SI refers to an acute, immediate or imminent risk requiring urgent psychiatric intervention, chronic SI was defined as experiencing suicidal ideation of varying intensity on a continuous or intermittent basis over a period of months to years, with the ongoing likelihood of a person considering a future attempt.

Participants with chronic suicidal thoughts were referred to OKTOS by their general practitioner or psychiatrist. All participants referred to the trial had a diagnosis of major depressive disorder with one or more comorbidities, including generalised anxiety disorder, bipolar disorder, post-traumatic stress disorder and obsessive-compulsive disorder. The principal investigator and consultant psychiatrist (AC) obtained a psychiatric history and assessed the mental state of participants, including severity and chronicity of their SI.

Participants referred to the trial were offered ketamine under the rationale of having exhausted alternate treatment options for chronic SI or having unsatisfactory treatment outcomes, including being unable to tolerate side effects of currently available treatment; all had failed to respond to at least two pharmacological interventions. Informed, written consent was obtained from individual participants. The trial was approved by the Bellberry Human Research Ethics Committee (# 2017–12-982) and approval ratified by the University of the Sunshine Coast Human Research Ethics Committee (# A181101). The 10-week trial period comprised a pre-treatment assessment, followed by a six-week ketamine treatment phase, and a four-week post-treatment follow-up phase.

### Inclusion and exclusion criteria

Participants aged 18 years or over with chronic suicidal thoughts as the primary presenting complaint, determined by the Beck Scale for Suicide Ideation (BSS) score ≥ 6 at screening, were included in the OKTOS trial. Participants were excluded from the trial if they were experiencing acute SI or had a history of psychosis, mania or hypomania. Full inclusion and exclusion criteria for OKTOS are shown in Table 1. The exclusion criteria developed for the OKTOS trial were derived from ketamine’s known, important side effects and align with previous ketamine studies. It is widely recognized that ketamine can cause psychotic, manic and hypomanic symptoms which are acute psychiatric conditions and by themselves can foster a risk of SI [33]. Ketamine can also potentially cause hepatotoxicity, with modest, temporary elevations in liver enzymes noted at anaesthetic doses [34]. Trials investigating the efficacy of sub-anaesthetic doses of IV ketamine for depression consistently report transient elevations in blood pressure and heart rate during the period of the infusion and for up to 80 min after dosing [35, 36].

**Table 1:** Inclusion and exclusion criteria for the Oral Ketamine Trial on Suicidality (OKTOS)

Inclusion criteria	Exclusion criteria
● Persons aged ≥18 years	● Psychiatric conditions: ○Psychosis ○Mania/hypomania ○Acute suicidal ideation requiring urgent psychiatric intervention
● Participants with chronic suicidal thoughts as the primary presenting complaint, determined by Beck Scale for Suicide Ideation – 21 items (BSS) score ≥ 6 at screening	● Physical conditions: ○ Uncontrolled/severe symptomatic cardiovascular disease states including recent myocardial infarction (within prior 6 months); history of stroke; and hypertension (resting blood pressure > 150/100) ○History of intracranial mass, intracranial haemorrhage/stroke, cerebral trauma/traumatic brain injury or increased intracranial pressure (as assessed by referring general practitioner) ○ liver function test results out of normal range, as specified: ▪alanine aminotransferase: > 135 U/L ▪aspartate aminotransferase: > 123 U/ L ▪gamma glutamyl transferase male participants: > 210 U/L ▪gamma glutamyl transferase female participants: > 135 U/L ▪total bilirubin > 60 umol/L ▪albumin: < 25 g/L and > 150 g/L ▪alkaline phosphatase: > 345 U/L
	● Previous reaction to ketamine (as reported by referring general practitioner and participant)
	● Pregnant women
	● Breastfeeding women

Participants underwent baseline assessment comprising physical examination, urinalysis, laboratory blood testing, and medical history, obtained by the principal investigator/consultant psychiatrist within 14 days prior to commencement of ketamine treatment. Baseline assessment also included administration of standardised clinical rating scales. During the treatment and follow-up phase, patients maintained their regular medication regimen as prescribed by their health professionals. Concurrent psychopharmacological agents taken by patients included antidepressants and mood stabilisers.

### Treatment

Participants received sub-anaesthetic doses of oral ketamine during the six-week treatment phase, at one dose per week. The initial dose of ketamine was 0.5 mg/kg, administered in fruit juice by the consultant psychiatrist. Dose amounts were flexible, titrated up by 0.2–0.5 mg/kg or down by 0.2–0.7 mg/kg at each treatment, depending on participant tolerance, with a maximum dose of 3.0 mg/kg at the sixth treatment. Participant tolerance to ketamine was determined by the presence or absence of side effects or discomfort, as measured by tolerability and safety rating scales, vital signs (blood pressure, pulse rate, oxygen saturation, temperature) and urinalysis. Patients were supervised by mental health nurses (MD, MJ) immediately post treatment and all were seen by the consultant psychiatrist before leaving the study premises. During the trial, patients were assessed via nine study visits and via eleven phone assessments between visits. Study visits occurred at ‘baseline’, once per week during treatment weeks 1–6 and then at two additional visits during the four-week follow-up phase. Phone assessments, undertaken by mental health nurses primarily to check for adverse effects, took place twice a week after treatments 1–5 and once after treatment 6.

**Fig. 1:**
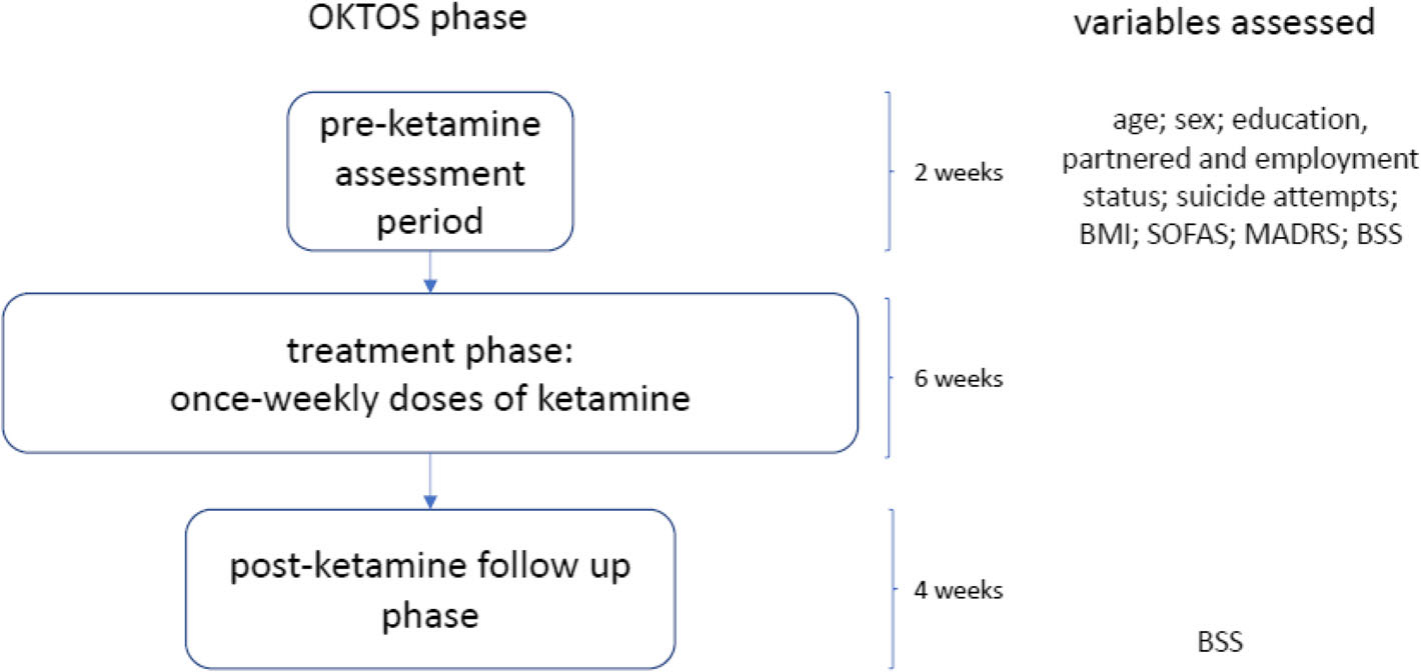
OKTOS treatment and assessment procedure. BMI = Body Mass Index; SOFAS = Social and Occupational Functioning Assessment Scale; MADRS = Montgomery-Asberg Depression Rating Scale; BSS = Beck Scale for Suicide Ideation

Vital signs (heart rate, blood pressure, oxygen saturation level and temperature) were recorded at the baseline assessment, as well as three times at the treatment visit - prior to ketamine administration and at 30- and 60-min post-treatment. Blood samples were taken at four timepoints: at baseline, at the third weekly treatment, just after the last treatment and at the end of the four-week follow-up phase. Serum hematological and biochemical parameters monitored included cholesterol, morning cortisol, brain-derived neurotropic factor, thyroid function, full blood count, liver function, urea and electrolytes. Urinalysis was performed at the pre-ketamine visit as well as prior to all treatments to screen for possible cystitis. Urine pregnancy screening was undertaken for females of childbearing age. Figure 1 illustrates the phases of the trial, including the timing of assessments relevant to the BN study.

### Prolonged treatment response

A prolonged therapeutic response to ketamine treatment was defined as a reduction in SI following a course of six treatments, defined by either (i) ⩾50% improvement in the pre-treatment BSS score at the end of the four-week post-treatment follow-up phase, or (ii) BSS score ≤ 6 at the end of the four-week post-treatment follow-up phase [37].

### Data collection and preparation

The clinical rating scales and demographic questions were administered to participants on a touch screen tablet, using the Qualtrics survey platform (Copyright© 2019 Qualtrics, Provo, UT, USA). The SPSS version 24.0 (IBM Corp 2016) was used for data preparation in conjunction with the Python programming package SciPy [38].

### Variables used in the BN

Variables from OKTOS used in the current study are summarised in Table 2. OKTOS variables were selected as inputs to the prolonged response prediction BN based on clinical experience or prior evidence in the literature of an association with ketamine response. One latent, discrete variable (*profile)* was incorporated in the model to prevent formation of very large conditional probability tables. In addition to the demographic variables presented in Table 2, three clinical rating scales included in the final BN structure are described here.

**Table 2:** Oral Ketamine Trial on Suicidality (OKTOS) variables used in BN for predicting therapeutic response to ketamine

OKTOS variable/s	BN node
Participant age	Age
Sex	Sex
Highest education level achieved	Tertiary education
Employment status	Employed
Marital status	Partnered
Number of previous suicide attempts	Suicide attempts
Weight in kg/(height in m)^2^ (pre-treatment)	BMI
n/a	Profile
MADRS (pre-treatment)	MADRS
SOFAS (pre-treatment)	SOFAS
BSS (end of follow-up phase)	Response

The *Social and Occupational Functioning Assessment Scale* (SOFAS), used to rate functioning for the current period, focuses exclusively on the individual’s level of social and occupational functioning and is not directly influenced by overall severity of the individual’s psychological symptoms [39]. The SOFAS is a one-item rating of functioning with a possible range of 0–100, where higher scores indicate higher social and occupational functioning. The SOFAS is considered to have superior predictive and concurrent validity in comparison to other commonly used measures [40] and has been regularly utilised in clinical populations, including those exhibiting suicidal ideation [41].

The *Beck Scale for Suicide Ideation* (BSS), a well-established clinician rating scale, is a 19-item clinical research instrument designed to assess and quantify the intensity, pervasiveness and characteristics of suicidal ideation in adults [42]. The BSS possible range is 0–38, with scores of ≥6 indicating clinically significant suicidal ideation. The BSS has been validated in male adults aged 19–75 years (*N* = 342), demonstrating strong correlation between suicide risk assessment and number of previous suicide attempts, with higher BSS scores relating to increased number of suicide attempts [43].

The *Montgomery-Asberg Depression Rating Scale* (MADRS), is a ten-item diagnostic questionnaire used by psychiatrists to measure the severity of patients’ depressive episodes [44]. The MADRS questionnaire includes questions on the following symptoms: apparent sadness, reported sadness, inner tension, reduced sleep, reduced appetite, concentration difficulties, lassitude, inability to feel, pessimistic thoughts, and suicidal thoughts. The possible range for MADRS scores is 0–60. The MADRS was validated for use in adults in a landmark study by Montgomery and Asberg and has demonstrated excellent inter-rater reliability. This study has been widely cited nationally and internationally [45].

### Model structure and parameterisation

An unparameterised causal network containing 11 variables was created by the OKTOS project team using GeNIe 2.3.3828.0 (© BayesFusion LLC, 2019). Demographic variables and pre-treatment clinical rating scales were used as inputs to the model. Input or predictor variables were observed in the pre-treatment phase of the trial and the outcome variable *prolonged response* was observed at the end of the four-week post-treatment follow-up phase of the trial. Binary states were chosen for all variables except *age*, *suicide attempts* and *BMI,* where finer granularity was considered important. Continuous variables were discretised using cut-offs from the literature, or uniform widths where no established cut-offs were available; categorical variables were discretised using cut-offs from the literature or thresholds determined by the study authors where established cut-offs were not available (Table 7 in Appendix). Parameter learning from the discretised data set was accomplished using the expectation-maximisation algorithm [46, 47], with randomised parameter initialisation. Once parameterised, model performance was reviewed and modified by mental health clinicians, in conjunction with the OKTOS project team, then validated by domain experts from the disciplines of psychiatry and neurobiology.

### Model validation

Evaluation of the model was performed using K-fold cross validation [48]. The confusion matrix generated in GeNIe assessed model accuracy as 97%, and the area under the receiver operating characteristic (ROC) curve (AUC) value was found to be 0.87; the model correctly predicted 15/15 responders and 14/15 nonresponders. Sensitivity analysis, described in the Results, completed the model validation procedures, in the absence of other Bayesian networks in this domain with which to compare model output.

### Scenarios - single or multiple variable evidence introduction

The parameterised BN was used to model the effects of single variable or multivariate ‘what-if’ scenarios. Evidence was introduced in a single variable by selecting a node state. Multivariate scenarios were modelled to demonstrate how the BN might be used to assist clinical decision making, using four fictional candidates for ketamine treatment for chronic SI. Single variable and multivariate scenarios are presented in the Results.

### Statistical analysis

Categorical variables were described in terms of frequencies and percentages. Continuous variables were summarised as means ± standard deviation (SD) after assessing for normality using the Shapiro-Wilk test. To study the effect of new evidence introduced into the network on the chosen outcome measures, the percent change (Δ_%_) in each response node state was calculated as

1 $$ {\Delta}_{\%}=\frac{P_{\mathrm{evidence}-}{P}_{\mathrm{baseline}}}{P_{\mathrm{baseline}}}\times 100\% $$

where *P*_baseline_ is the probability of occurrence of response node states under baseline network conditions before new evidence is introduced and *P*_evidence_ is the probability of a state occurring after new evidence is introduced into the network.

## Results

### General characteristics of the study population

Of the 64 participants screened, 40 participants (63%) met the inclusion criteria for OKTOS. Of these, five were withdrawn due to clinical concerns and three elected not to continue with treatment. A total of 32 participants completed 6 weeks of ketamine treatment, although of these, two participants were not available for follow-up assessment four-weeks after treatment cessation. Participants had a mean (SD) age of 45.7 (14.2) years (min 22.2 years, max 71.8 years) and mean (SD) weight at first treatment of 91.1 kg (24.8) kg (min 56 kg, max 154 kg). Twenty-seven participants (84%) had a medical comorbidity. All participants met *Diagnostic and Statistical Manual of Mental Disorders* (Fifth Edition; 3) criteria for major depressive disorder and were experiencing chronic SI without psychotic features at the time of commencing the trial. Summary data used in the current study are summarised in Table 3.

**Table 3:** Summary data from Oral Ketamine Trial on Suicidality (OKTOS) used in BN for predicting therapeutic response to oral ketamine (*n* = 32 at pre-treatment assessment phase)

Variable	n	%
Age		
<25 years	2	6
25–44 years	13	41
45–64 years	15	47
≥ 65 years	2	6
Female	16	50
Tertiary education	19	59
Employed	10	31
Partnered	13	41
Suicide attempts		
none	7	22
one	9	28
two or more	16	50
BMI		
healthy	11	34
overweight	7	22
obese	14	44
MADRS		
not severe	4	13
severe	28	87
SOFAS		
low	11	34
high	21	66
Prolonged response		
yes	15	50^a^
no	15	50^a^

^a^*n* = 30 at follow-up phase

### Treatment outcomes

At follow-up, 15 of 30 participants (50%) were assessed as being responders (defined as ≥50% improvement from pre-treatment BSS, or BSS ≤ 6 at the end of the four-week post-ketamine follow-up phase. Two participants were lost to follow-up. Of the two criteria used to delineate responders, 15 participants (50%) showed ⩾50% improvement in the pre-treatment BSS score, and 12 participants (40%) had BSS scores ≤6 at the end of the follow-up period (Table 4). Ten participants (33%) were assessed as being in full remission (defined as BSS = 0) at the end of the 10-week trial period.

**Table 4:** Concurrence of two study response criteria (*n* = 30)

		Criterion 2	
		yes	no
Criterion 1	yes	12 (40%)	3 (10%)
	no	0 (0%)	15 (50%)

Criterion 1 = ≥ 50% improvement from pre-treatment BSS at end of 4-week post-ketamine follow-up phase

Criterion 2 = BSS ≤ 6 at end of 4-week post-ketamine follow-up phase; 2 participants lost to follow-up

### Baseline network and sensitivity assessment

**Fig. 2:**
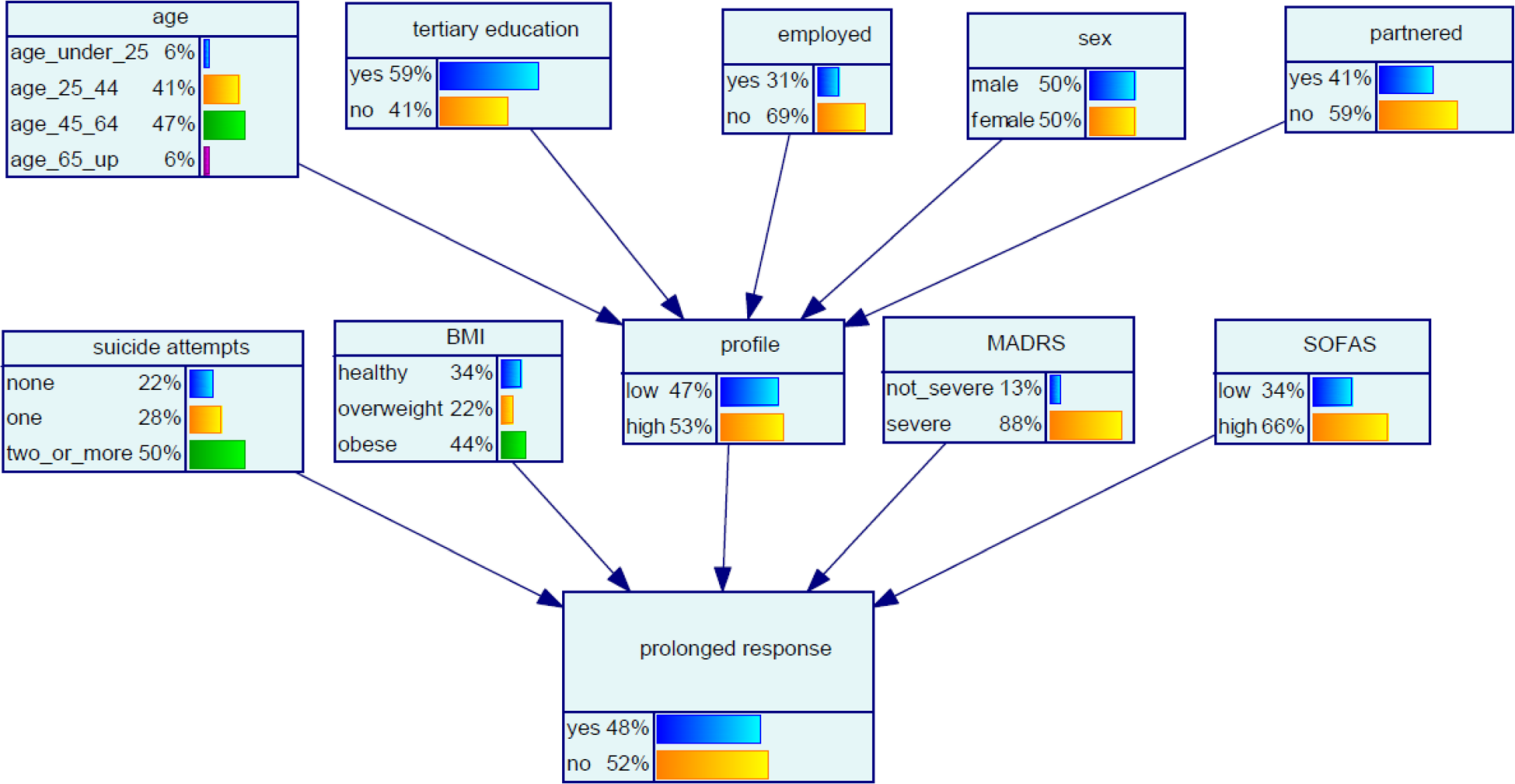
Bayesian network (BN) for predicting prolonged ketamine response in chronic SI. Predictor variables were observed in the pre-treatment assessment phase, response variable was observed at the end of the post-treatment follow-up phase

The parameterised BN at baseline (i.e., with no evidence introduced) is shown in Fig. 2.

Sensitivity analysis of model priors was conducted using an algorithm proposed by Kjaerulff and van der Gaag [49] to give an indication of the relative importance of model inputs in terms of precision. The assessment showed that, in a network with no introduced evidence, the target node *prolonged response* was most sensitive to *BMI*, followed by the *SOFAS, MADRS, suicide attempts, employed, age* and *tertiary education* nodes. Table 5 shows the sensitivity of the *prolonged response* node to all input variables, in rank order of variables with most impact on *prolonged response.*

**Table 5:** Prior (no new evidence) sensitivity of *prolonged response* node to input nodes in order of decreasing sensitivity of the *prolonged response* node to inputs. Table shows maximum sensitivity analysis derivatives [49]

Input node to ***prolonged response***	Maximum sensitivity analysis derivative^**a**^
BMI	0.182
SOFAS	0.171
MADRS	0.116
Suicide attempts	0.105
Employed	0.067
Age	0.043
Tertiary education	0.024
Sex	0.014
Partnered	0.008

^a^Kjaerulff and van der Gaag [49]

In the next section we present four fictional candidates for ketamine treatment for chronic SI, modelling their presenting characteristics as scenarios in the BN.

### Scenarios

#### Scenario 1 ‘Tiffany’

**Fig. 3:**
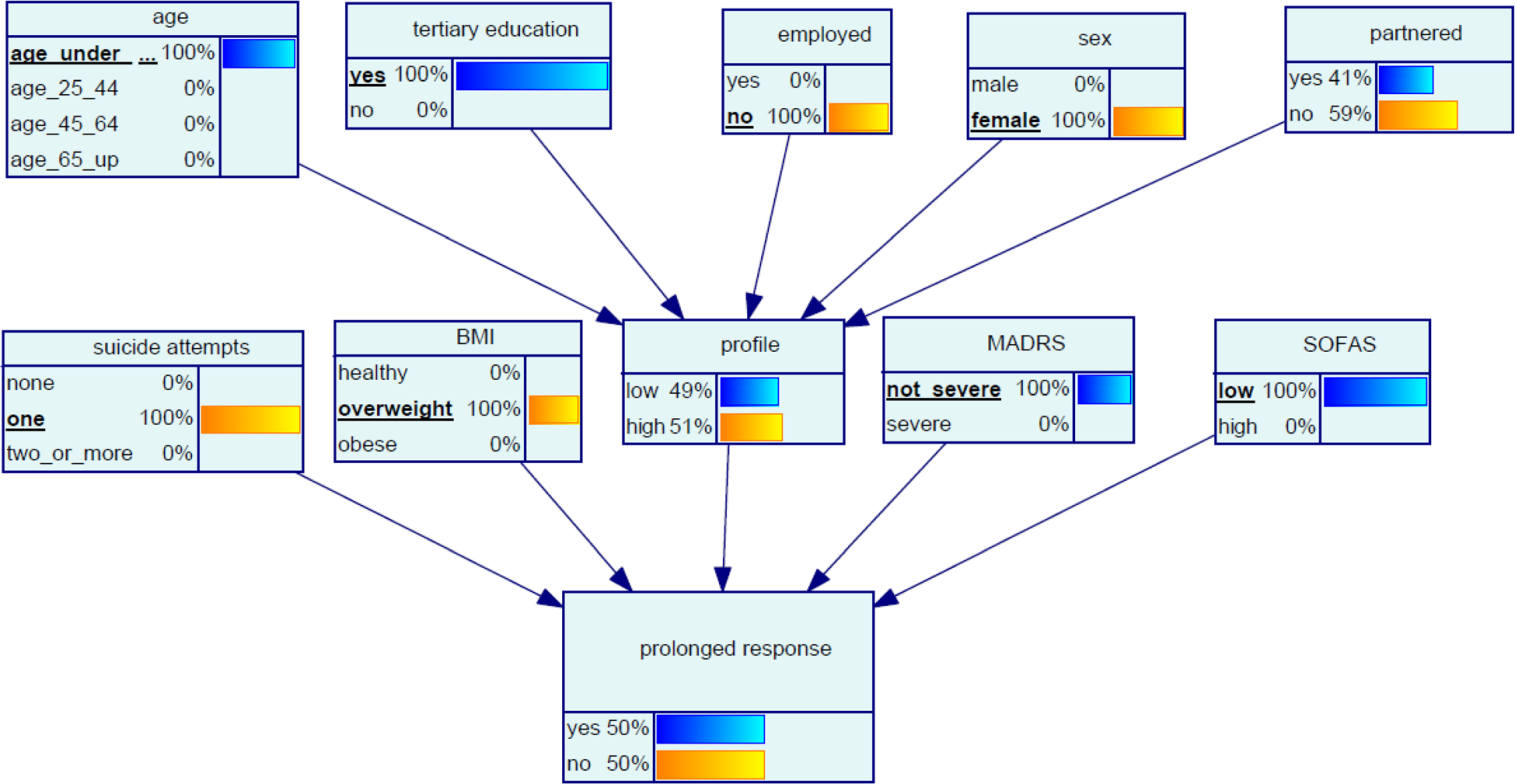
Bayesian network (BN) for predicting chance of response to oral ketamine for Scenario 1 ‘Tiffany’

Tiffany, aged 23, has not been able to find a job since finishing her university degree. Tiffany’s BMI indicates she is overweight, and she has attempted suicide once. Her SOFAS is low and her MADRS score indicates her depression is in the moderate range. The BN model indicates that the chance of Tiffany having a prolonged response to oral ketamine is 50% (Fig. 3).

#### Scenario 2 ‘Brianna’

**Fig. 4:**
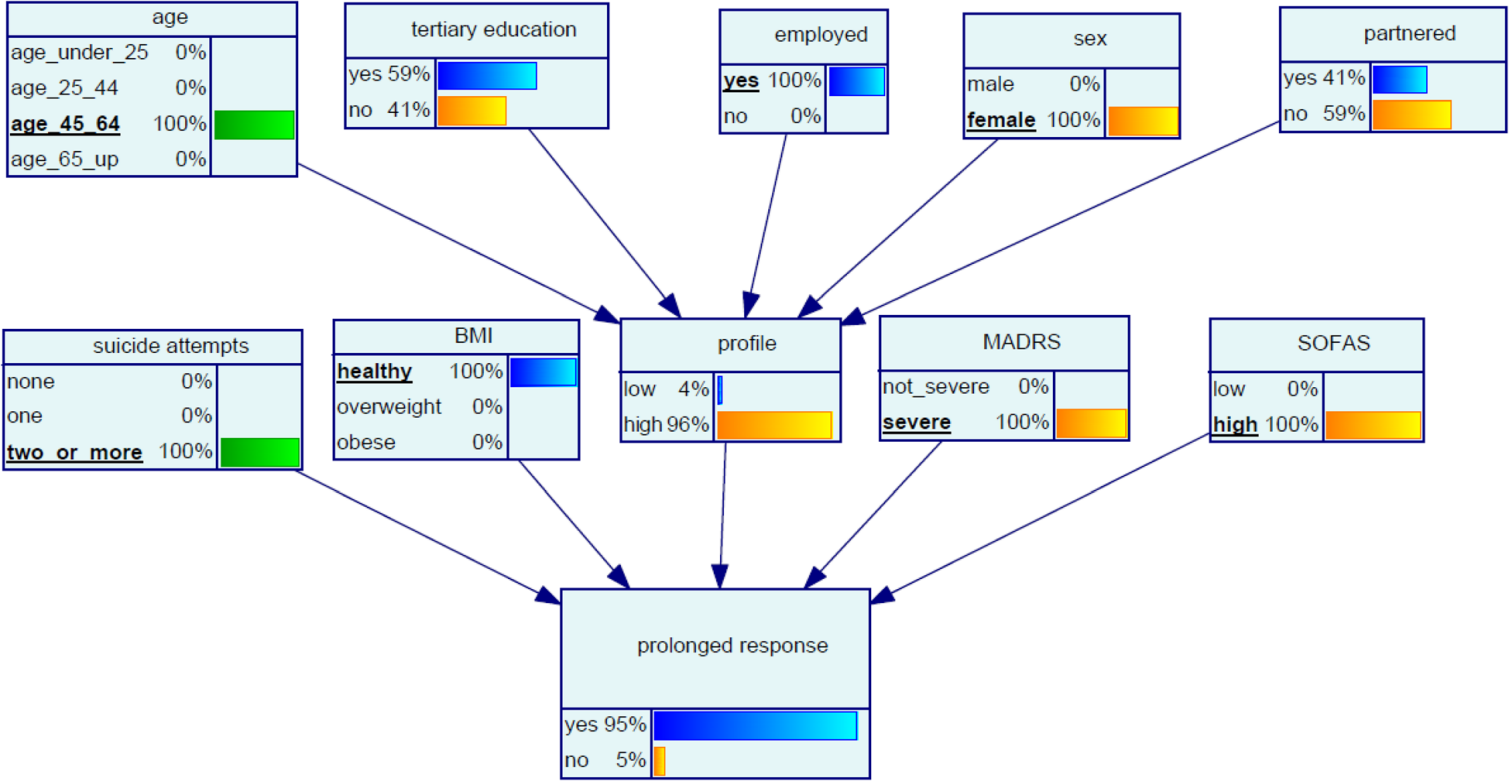
Bayesian network (BN) for predicting chance of prolonged response to oral ketamine for Scenario 2 ‘Brianna’

Brianna, aged 45, has a long history of depression and chronic SI and has attempted suicide several times. Brianna’s MADRS indicates her depression is severe. She is very fit as she exercises regularly, and her BMI is in the healthy range. Brianna is working part time as a casual and reports good relationships with her co-workers and family – her SOFAS is high. The model indicates that the chance of Brianna having a prolonged response to ketamine is 95% Fig. 4.

#### Scenario 3 ‘Levi’

**Fig. 5:**
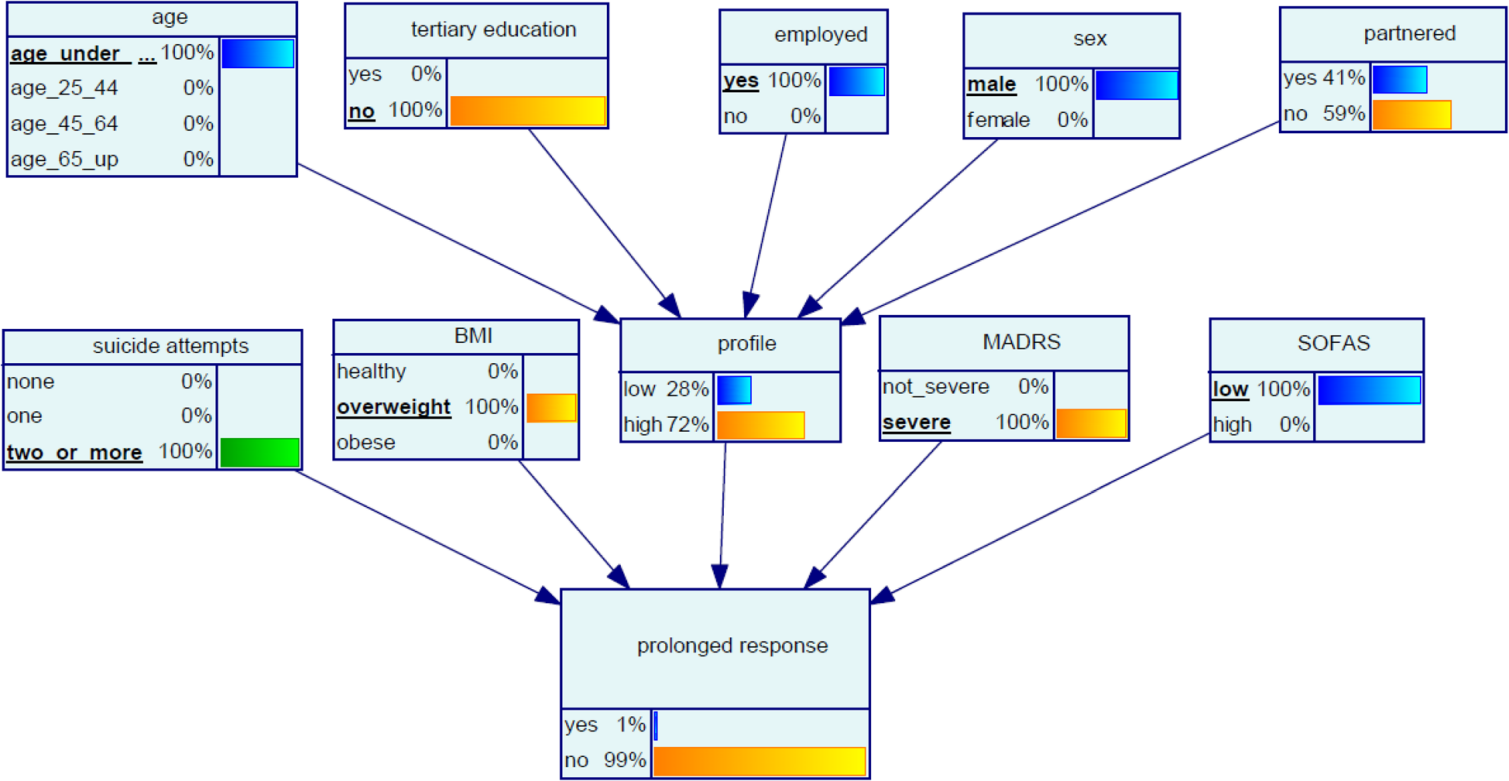
Bayesian network (BN) for predicting chance of prolonged response to oral ketamine for Scenario 3 ‘Levi’

Levi, aged 19, left school at 14. He has recently moved towns to live with his grandmother. He works on a casual basis at a fast food outlet and is overweight. His relocation has exacerbated his depression and his MADRS indicates severe depression and his SOFAS is low. He has a history of several suicide attempts. The model suggests that Levi’s chance of prolonged response to ketamine treatment is 1% (Fig. 5).

#### Scenario 4 ‘Kamal’

**Fig. 6:**
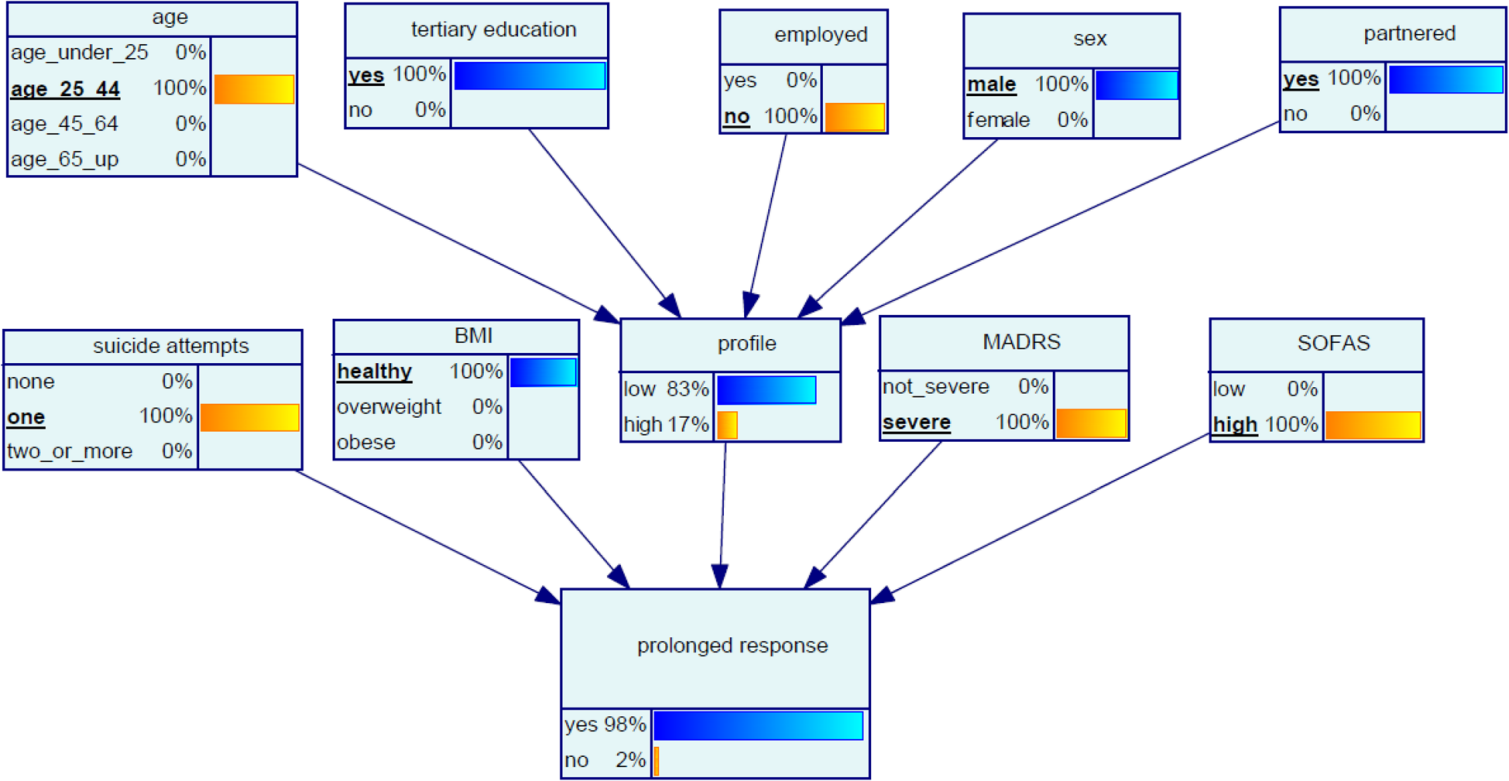
Bayesian network (BN) for predicting chance of prolonged response to oral ketamine for Scenario 4 ‘Kamal’

Kamal, aged 39, married, is from a non-English speaking background and has a tertiary education. Kamal has been unable to find a job using his qualifications since coming to Australia. He has been referred with a history of chronic SI and has attempted suicide once. Kamal’s MADRS indicates his depression is severe, and his SOFAS score is 57. Kamal’s BMI is in the healthy category. The model suggests that the likelihood of Kamal having a prolonged response to oral ketamine is 98% (Fig. 6).

### Evidence entered in single nodes

In addition to the multivariate scenarios, we examined the effect of evidence entered in single predictor nodes on the outcome variable *prolonged response* (Table 6). Participants were more likely to have a prolonged response to ketamine if they were male, aged under 25 years, had no tertiary education, were not partnered, had no previous suicide attempts and had a healthy BMI. Participants were also more likely to respond if they had a ‘high’ SOFAS (i.e. > 55) or a MADRS score in the range 0 to 34 (i.e. not severe) at their pre-treatment assessment. Significantly, whether evidence entered in a single node increased or decreased the likelihood of a positive therapeutic response varied according to the combinations of evidence in other nodes. For example, although not having a tertiary education increased the chance of prolonged response by 4% in this sample (Table 6), when combined with evidence described in Scenario 2, not having a tertiary education *decreased* Brianna’s chance of a prolonged response to ketamine from 95 to 91% (a decrease of 4%).

**Table 6:** Chance of response to ketamine as a result of evidence entry in single nodes – BN for predicting likelihood of prolonged response to oral ketamine. Baseline chance of prolonged response (no evidence entered) = 48%, baseline chance of no prolonged response = 52%

Single state scenario	% chance of prolonged response (Δ_%_)^**a**^	% chance of no prolonged response (Δ_%_)^**a**^
Age < 25 years	50 (4)	50 (− 4)
Age 25–44 years	46 (− 4)	54 (4)
Age 45–64 years	50 (4)	50 (− 4)
Age ≥ 65 years	47 (− 2)	53 (2)
Sex male	49 (2)	51 (− 2)
Sex female	48 (0)	52 (0)
Tertiary education	47 (−2)	53 (2)
No tertiary education	50 (4)	50 (−4)
Employed	53 (10)	47 (−10)
Not employed	46 (−4)	54 (4)
Partnered	48 (0)	52 (0)
Not partnered	49 (2)	51 (−2)
No previous suicide attempts	57 (19)	43 (−17)
One previous suicide attempt	50 (4)	50 (−4)
Two or more previous suicide attempts	44 (−8)	56 (8)
BMI healthy	57 (19)	43 (−17)
BMI overweight	34 (−29)	66 (27)
BMI obese	49 (2)	51 (−2)
MADRS not severe	59 (23)	41 (−21)
MADRS severe	47 (−2)	53 (2)
SOFAS low	37 (−23)	63 (21)
SOFAS high	54 (13)	46 (−12)

^a^ $$ {\Delta}_{\%}=\frac{P_{\mathrm{evidence}-}{P}_{\mathrm{baseline}}}{P_{\mathrm{baseline}}}\times 100\% $$

## Discussion

This study sought to develop a practical method of predicting likelihood of prolonged response to treatment with serial doses of oral ketamine for chronic SI. Using BN methodology, we evaluated a combination of demographic variables and pre-treatment clinical measures to predict likelihood of prolonged therapeutic ketamine response at the end of the post-treatment follow-up period. Through use of multivariate scenarios, we demonstrated that employing demographic and clinical rating scale variables in a BN provides rich insights into networks of factors predicting likelihood of prolonged response to ketamine. The variables age, sex, employment and partnered status, level of education, number of previous suicide attempts, BMI, depression symptoms and social and occupational functioning appeared to be key predictor variables for prolonged response to oral ketamine in the OKTOS trial.

Single variable modelling of the OKTOS data showed that ketamine treatment candidates were more likely to have a prolonged response to treatment if they were male, aged under 25 years, had no tertiary education, were not partnered, had no previous suicide attempts, a healthy BMI, a non-severe MADRS score and a high SOFAS score. The model did not predict high likelihood of prolonged response to oral ketamine in those who had had two or more previous suicide attempts; the single variable scenarios predicted improved chance of prolonged response for participants with no or one previous suicide attempt and decreased chance of response for participants with two or more previous attempts. However, suicide risk is not contingent solely on the history of previous attempts, but is influenced by a number of interacting factors, and our model endeavours to reflect some of the factors we believe mitigate and compound both the risk, and response to the treatment. The relationship between the number of previous attempts and suicide risk warrants further investigation in future studies.

In exploring evidence introduction in single nodes further, we demonstrated that regardless of model response to evidence introduction in a single node, the same evidence in a multivariate scenario could increase or decrease likelihood of response, contingent on the combination of evidence in other nodes. This is an elegant illustration of the efficiency of the BN framework in modelling the mutual distribution of all states in all nodes in the network, considering node dependencies and any new evidence introduced to the network simultaneously. In practical terms, this representation of the joint probability distribution of the network demonstrates the utility of the BN in twenty-first century clinical decision support, where the volume of available data exceeds not only the capabilities of traditional analytical approaches, but also of human cognition.

An important finding that emerged was the target variable *prolonged response* was most sensitive to *BMI,* of all variables in the model (Table 5). Closer examination of model behaviour with respect to BMI inputs without evidence introduced in other nodes, revealed that a BMI in the healthy or obese range increased the chance of an individual demonstrating a prolonged therapeutic response to ketamine by 19 and 2%, respectively (Table 6). However, the model indicated that a BMI in the overweight range decreased an individuals’ chance of responding by 29% and increased their chance of no prolonged response by 27%. Our results for both healthy and overweight BMI are inconsistent with a previous study by Niciu et al. (2014) [17], showing that a higher BMI was associated with greater response to IV ketamine; although the endpoint in that study was antidepressant response, as opposed to an endpoint of reduction in SI, as was the case in our study. Niciu et al. (2014) observed that the patients receiving the highest ketamine dose (in mg) had greater improvements in their Hamilton Depression Rating Scale scores, suggesting that the association between BMI and acute antidepressant response might be a secondary effect of a relatively higher administered dose of ketamine. No significant association was found in OKTOS between higher doses of ketamine and greater improvements in either the BSS or the MADRS. Based on results of a subsequent study, the same researchers [50] suggested that systemic metabolic abnormalities might predict positive response to ketamine or at least have an indirect role in its therapeutic effects. These results were upheld in a study by Dale, Bryant [51], who concluded that BMI was not a predictor of response to ketamine, and outcome in antidepressant therapy was influenced by the presence of metabolic syndrome rather than obesity itself.

In artificial intelligence and Bayesian statistics, the principle of Occam’s Razor is often used to simplify models, where the simplest solution is often the best one [52]. Development of this model was an iterative process, with varying permutations of variables, guided by the domain knowledge of mental health clinicians. However, parsimony is a primary goal in BN design, where the simplest structure should be used to describe the system under consideration [53]. Variables such as medication class (antidepressants, antipsychotics, mood stabilisers), the DASS21 (Anxiety), DASS21 (Stress) and DASS21 (Depression) subscales, and a dichotomous indicator for persistent pain were originally included in the model, however while these variables were surmised to be clinically relevant, they reduced the sensitivity of the network and were able to be excluded without forfeiting model accuracy.

According to the National Institutes of Health, precision medicine is an emerging approach for disease treatment and prevention “that takes into account individual variability in genes, environment, and lifestyle for each person” [54]. Precision medicine approaches allow doctors and researchers to predict more accurately which treatment and prevention strategies for a disease will work in which groups of people. The decision to use ketamine to combat chronic suicidal ideation is currently made by the clinician in collaboration with the patient. Systematic, evidence-based identification of pre-treatment predictors of response to ketamine in chronically suicidal patients may streamline such decision making for clinicians, by empirically establishing the phenotype most likely to benefit from ketamine. The ability of BN to present and evaluate multiple paradigms is particularly appealing in understanding complex systems and they have the capacity to augment clinician judgement as probabilistic decision support systems in psychiatry and medicine.

### Limitations

This study yielded a proof of concept BN for predicting ketamine response in chronic SI and has the following methodological limitations. First, the findings cannot be extrapolated to other populations without external validation on data sets other than the one used to develop the model, including utilisation in populations with acute SI. Second, the sample size which the BN was based upon was relatively small (*n* = 32), although technically, there is no minimum sample size for BN [55]; they have been shown to have good predictive accuracy with small sample sizes [55, 56], and Bayesian methods are often selected over frequentist methods to better accommodate reduced sample sizes [57]. However, these findings should be treated with some caution and future studies with larger sample sizes that replicate these analyses are warranted. Third, the data used in the model were from a secondary source, and some of the variables used to predict ketamine response in the literature (e.g., family history of alcohol use disorder, indicators of cognitive functioning) were not available for inclusion in the model. Nevertheless, the model created using OKTOS variables appears to provide accurate predictions. Fourth, the definition of therapeutic response used included ≥50% improvement in the pre-treatment BSS. This cut-off value was arbitrarily selected to define response, however the extent to which this threshold accurately reflects therapeutic changes in SI is unknown. Lastly, the short post-ketamine follow-up period (4 weeks) was a limitation of the OKTOS trial, particularly as short follow-up for treatment efficacy is particularly unreliable for chronic conditions.

### Further research

BN are an iterative modelling tool, and here we present a first iteration of a model which can potentially be developed with further data and wider consultation, to form a component in a desktop decision support system for clinicians. In this study, model inputs are pre-treatment measures - further model development could incorporate clinical ratings gathered during the treatment phase. Future iterations of the BN described here could also include tolerability and safety measures, including dissociative phenomena, cystitis and urinary incontinence; and other variables such as pre-treatment biochemistry (e.g., brain-derived neurotrophic factor), neurochemistry (e.g., glutamine/glutamate ratio), neuroimaging (e.g., anterior cingulate cortex activity), and cognitive functioning (i.e., processing speed). In other variations of the model, discretisation of clinical rating scales could be undertaken using expert elicitation with mental health clinicians. Finally, given the efficacy of oral ketamine over a period of 6 weeks, future studies should examine the longer-term effects including the efficacy of ketamine treatment over longer periods of time, including designs with less frequent dosing. The BN model developed here could undergo evaluation in subsequent clinical trials of ketamine, comparing model-predicted and actual response rates.

## Conclusion

Ketamine appears to be a promising treatment for people with chronic SI, however further investigation to determine clinical utility on an individual basis is necessary. As we have shown, use of standard demographic data and commonly used clinical rating scales in a BN model can provide an objective synthesis of factors impacting likelihood of prolonged response to ketamine in individuals with chronic SI, supporting clinical decision making. Significantly, obtaining BMI, sociodemographic information and history of suicide attempts are all part of psychiatric history taking, and the clinical rating scales used in this model are frequently used to monitor individuals’ clinical status during treatment. Finding simple, feasible predictors of therapeutic response to treatment is an important step towards improving the care of patients with chronic SI, in a context of suicide prevention.

## Data Availability

The datasets generated and/or analysed during the current study are not publicly available for reasons of participant confidentiality but are available from the corresponding author on reasonable request.

## Appendix

**Table 7:** Thresholds and definitions for node states – Bayesian network for prediction of prolonged oral ketamine response

Variable	Definitions and thresholds for states	Discretisation method/ source
Age	age_under_25 = < 25 years	determined by study authors
	age_25_44 = 25 ≤ years < 45	
	age_45_64 = 45 ≤ years < 65	
	age_65_up = ≥ 65 years	
Sex	male	determined by study authors
	female	
Tertiary education	yes = tertiary education	determined by study authors
	no = no tertiary education	
Employed	yes = employed; full time, part time or casual	determined by study authors
	no = unemployed or not in labour force	
Partnered	yes = married/de facto	determined by study authors
	no = never married, widowed, divorced, separated	
Suicide attempts	none = no previous suicide attempt	determined by study authors
	one = one previous suicide attempt	
	two_or_more = ≥ two previous suicide attempts	
BMI	underweight = < 18.5	[58]
	healthy = 18.5–24.9	
	overweight = 25–29.9	
	obese = ≥ 30	
Profile	low, high; latent variable (unobserved, parameterised during automated learning)	not applicable; latent variable
MADRS	not_severe = ≤ 34	[59]
	severe = > 34	
SOFAS	low = < 55	uniform widths
	high = ≥ 55	
Prolonged response	yes = (i) ⩾50% improvement from pre-treatment BSS at the end of the post-treatment follow-up phase, or (ii) BSS score ≤ 6 at the end of the post-treatment follow-up phase no = no improvement as defined for ‘yes’	determined by study authors

## Abbreviations

AUC: Area under the curve
BMI: Body mass index
BN: Bayesian network
BSS: Beck Scale for Suicide Ideation
DASS21: Depression, Anxiety and Stress Scale (21 items)
IV: Intravenous
MADRS: Montgomery-Asberg Depression Rating Scale
OKTOS: Oral Ketamine Trial on Suicidality
ROC: Receiver operating characteristic
SD: Standard deviation
SI: Suicidal ideation
SOFAS: Social and Occupational Functioning Assessment Scale
